# Mycobacteria-responsive sonic hedgehog signaling mediates programmed death-ligand 1- and prostaglandin E_2_-induced regulatory T cell expansion

**DOI:** 10.1038/srep24193

**Published:** 2016-04-15

**Authors:** Sahana Holla, Emmanuel Stephen-Victor, Praveen Prakhar, Meenu Sharma, Chaitrali Saha, Vibha Udupa, Srinivas V. Kaveri, Jagadeesh Bayry, Kithiganahalli Narayanaswamy Balaji

**Affiliations:** 1 Department of Microbiology and Cell Biology, Indian Institute of Science, Bangalore, Karnataka, India; 2 Institut National de la Santé et de la Recherche Médicale, Unité 1138, Paris, France; 3 Centre de Recherche des Cordeliers, Equipe-Immunopathology and therapeutic immunointervention, Paris, France; 4 Sorbonne Universités, UPMC Univ Paris 06, UMR S 1138, Paris, France; 5 Université de Technologie de Compiègne, Compiègne, France; 6 Université Paris Descartes, Sorbonne Paris Cité, UMR S 1138, Paris, France

## Abstract

CD4^+^CD25^+^FoxP3^+^ regulatory T cells (Tregs) are exploited by mycobacteria to subvert the protective host immune responses. The Treg expansion in the periphery requires signaling by professional antigen presenting cells and in particularly dendritic cells (DC). However, precise molecular mechanisms by which mycobacteria instruct Treg expansion via DCs are not established. Here we demonstrate that mycobacteria-responsive sonic hedgehog (SHH) signaling in human DCs leads to programmed death ligand-1 (PD-L1) expression and cyclooxygenase (COX)-2-catalyzed prostaglandin E_2_ (PGE_2_) that orchestrate mycobacterial infection-induced expansion of Tregs. While SHH-responsive transcription factor GLI1 directly arbitrated COX-2 transcription, specific microRNAs, miR-324-5p and miR-338-5p, which target PD-L1 were downregulated by SHH signaling. Further, counter-regulatory roles of SHH and NOTCH1 signaling during mycobacterial-infection of human DCs was also evident. Together, our results establish that *Mycobacterium* directs a fine-balance of host signaling pathways and molecular regulators in human DCs to expand Tregs that favour immune evasion of the pathogen.

CD4^+^CD25^+^FoxP3^+^ regulatory T cells (Tregs) are one of the subsets of CD4^+^ T helper cells that are necessary for immune homeostasis. Apart from their roles in maintaining peripheral tolerance, Tregs have been heavily implicated for regulating immune responses against the invading pathogens^1^,^2^,^3^. Exhibiting contrast functions, Tregs can not only suppress protective immune responses and the collateral damage caused due the excessive inflammation during infections, but also provide suitable niche for facilitating the persistence of chronic infections such as tuberculosis^4^,^5^,^6^. Thus, induction and expansion of Tregs mark as one of the immune evasion strategies of mycobacteria for their survival in the host.

During mycobacterial infections, accumulation and proliferation of Tregs at the site of infection contributes to inhibition of bacterial clearance^5^,^7^ as well as inhibition of antigen-specific protective responses exhibited by γδ T cells^8^. Further, patients with active tuberculosis have increased population of circulating Tregs that suppress IFN-γ production by Th1 cells^9^. Correspondingly, *ex vivo* depletion of Tregs from the PBMCs alleviates the IFN-γ production in response to mycobacterial antigens^10^. While *in vivo* depletion of CD25^+^ T cells in mice enhanced the IFN-γ production, adoptive transfer of CD25^+^ T cells to mice infected with *M. tuberculosis* facilitates bacterial survival^11^. Thus, understanding the mechanisms that define such Treg-mediated survival strategies of mycobacteria is important.

Investigations in both murine and human models of tuberculosis have identified several mechanisms of Treg generation and expansion^12^,^13^. Of current interest, studies have highlighted the roles for PD-L1 (B7-H1/CD274) and COX-2-catalyzed PGE_2_ during mycobacteria-induced Treg induction and expansion. While PD-L1 deficient mice were increasingly sensitive to tuberculosis infection^14^, studies on human DCs showed that infection-induced PD-L1 was essential for expansion of Tregs^15^,^16^. Though PD-L1 KO mice exhibited elevated CD4^+^ T and CD8^+^ T cell responses, PD-1 expression was higher in CD4^+^ T cells in the PD-L1 KO mice, suggesting a possible suppression of PD-1 by PD-L1. Further, possibly due to chronic activation of immune cells and inflammation, PD-L1 KO mice exhibited increased mycobacterial CFUs in lung and death of mice^14^. Likewise, PGE_2_-responsive human Treg expansion was found during mycobacterial infection^17^. However, the mechanisms that mediate the expression of the molecules like PD-L1 and COX-2 in DCs are not established.

In this context, it is well constituted that mycobacterial infection of the cells instigates a plethora of signaling pathways that ultimately regulate the immune mediators to determine cell-fate decisions and outcome/s of the infection. Previous investigations from our laboratory implicates the roles for SHH, WNT, NOTCH1 and PI3K signaling pathways in modulating macrophage^18^,^19^,^20^,^21^ and DC^22^,^23^,^24^ responses. Further, NOTCH^25^,^26^, WNT^27^,^28^, and PI3K^29^,^30^ pathways were entailed to regulate the DC functions. Thus, we explored the roles for these signaling pathways in modulating the mycobacteria-induced Treg expansion and functions.

Here we demonstrate that infection-responsive activation of SHH-PI3K-mTOR-NF-κB signaling in human DCs was necessary for *M. bovis* BCG-induced Treg expansion. On the other hand, NOTCH signaling hindered the ability of the infected DCs to expand Tregs while the contribution of WNT signaling was not evident. Although no apparent influence of SHH and NOTCH1 signaling on DC phenotype in terms of the maturation markers HLA-DR, CD40, CD83, CD80 and CD86 was observed, pro-inflammatory cytokines such as TNF-α, IL-2, IL-1β and IL-6 were moderately NOTCH1-responsive and suppressed by SHH signaling. Further, experiments utilizing pharmacological inhibitors and conventional siRNAs indicated that both PD-L1 and COX-2/PGE_2_ were induced in DCs upon stimulation with *M. bovis* BCG and *M. tuberculosis* and were regulated by SHH signaling. While SHH-responsive transcription factor, GLI1 arbitrated COX-2 expression, mycobacteria-stimulated SHH signaling was found to suppress miR-324-5p and miR-338-5p, bonafide miRNAs that target PD-L1, to aid increased expression of PD-L1 and Treg expansion. Interestingly, inhibition of NOTCH1 signaling resulted in elevated expression of infection-induced PD-L1 whereas inhibition of SHH signaling showed increased transcripts of *JAG2* and NICD, markers for NOTCH activation. These results establish the mechanism of Treg expansion during mycobacterial infections, a testimony of its survival capabilities in the host.

## Results

### SHH and NOTCH signaling regulate *M. bovis* BCG-induced Treg expansion

**Figure 1:**
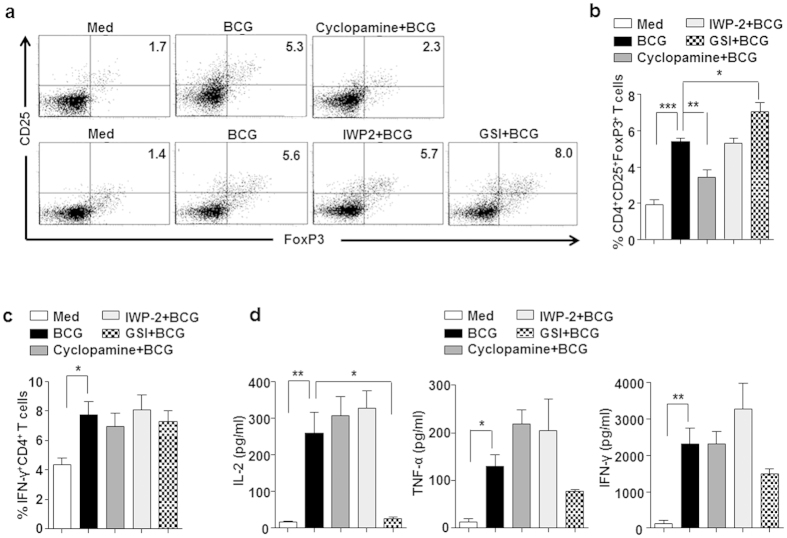
Opposing role of SHH and NOTCH1 signaling during *M. bovis* BCG-induced Treg expansion. (**a,b**) Immature DCs were cultured in GM-CSF and IL-4 alone (Med) or along with pharmacological inhibitors of SHH signaling pathway like Cyclopamine (SMO inhibitor), WNT signaling pathway like IWP-2 (a WNT secretion inhibitor) or NOTCH signaling like γ-secretase inhibitor (GSI) for 1 h followed by infection with *M. bovis* BCG (MOI 1:10) for 24 h. After extensive wash, DCs were co-cultured with autologous CD4^+^ T cells. CD4^+^CD25^+^FoxP3^+^ Tregs were analyzed by flow cytometry. (**a**) Representative dot blot of 6 independent experiments is shown. (**b**) Percentage of CD4^+^CD25^+^FoxP3^+^ cells in the DC-CD4^+^ T cell co-cultures (mean ± SEM, n = 8). (**c**) In a similar set up as panels (**a**,**b)** percentage of IFN-γ^+^CD4^+^ cells in the DC-CD4^+^ T cell co-cultures (mean ± SEM, n = 7). (**d**) T cell cytokines IL-2, TNF-α and IFN-γ were analyzed in the cell-free culture supernatants of DC:T cell co-culture (mean ± SEM, n = 6–7) by cytokine bead array. Med, Medium. *P < 0.05; **P < 0.005; ***P < 0.001 (one-way ANOVA followed by Turkey’s multiple-comparisons test).

**Figure 2:**
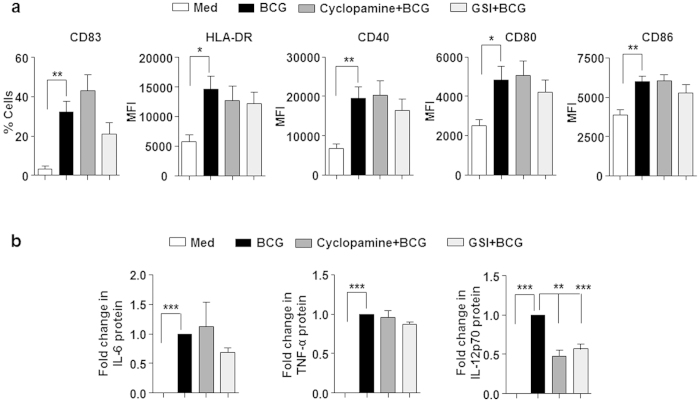
*M. bovis* BCG-induced proinflammatory responses are moderately NOTCH dependent and suppressed by SHH. DCs were cultured with GM-CSF and IL-4 and left untreated (Med) or infected with *M. bovis* BCG alone or after 1 h pretreatment with the indicated inhibitors for 24 h. (mean ± SEM, n = 6–10) (**a**) Surface expression of maturation markers CD83, HLA-DR, CD40, CD86 and CD80 were examined by flow cytometry. Data is represented as % positive cells or MFI. (**b**) Cell-free supernatants from the above-said experiment were assessed for secretion of IL-6, TNF-α, IL-12p70 by cytokine bead array (mean ± SEM, n = 5). Med, Medium. *P < 0.05; **P < 0.005; ***P < 0.001 (one-way ANOVA followed by Turkey’s multiple-comparisons test).

To investigate the molecular circuitry regulating mycobacteria-mediated Treg expansion, role for signaling pathways like NOTCH, WNT and SHH were assessed. Twenty-four hours post-infection with *M. bovis* BCG, DCs were washed and co-cultured with autologous CD4^+^ T cells for 5 days to analyze the expansion of Treg population. As shown in Fig. 1a,b, *M. bovis* BCG-infected DCs induced significant expansion of CD4^+^CD25^+^FoxP3^+^ Treg cells. However, DCs pretreated with cyclopamine (SHH pathway inhibitor) failed to induce Treg expansion. Further, treatment of DCs with GSI (NOTCH signaling activation inhibitor) enhanced their ability to expand Tregs. Interestingly, no significant difference was observed on perturbation of WNT pathway using IWP-2 (a WNT secretion inhibitor). These results suggest that SHH signaling positively regulates *M. bovis* BCG-mediated CD4^+^CD25^+^FoxP3^+^ Treg expansion and on the contrary, NOTCH signaling was found to exhibit negative regulation. Importantly, no definite roles for SHH and NOTCH signaling was found in mediating Th1 cell cytokine IFN-γ on *M. bovis* BCG infection (Fig. 1c,d). However, NOTCH signaling was required for infection-induced IL-2 (Fig. 1d). These data thus suggest selective role of SHH and NOTCH signaling pathways in modulating human Treg responses without disturbing IFN-γ responses. In line with these observations, perturbation of SHH and NOTCH signaling did not directly modulate either *M. bovis* BCG-induced DC maturation (Fig. 2a), or infection-stimulated secretion DC cytokines like TNF-α and IL-6 (Fig. 2b). Interestingly, infection-induced Th1-polarizing IL-12p70 was found to be SHH and NOTCH signaling dependent. Together, results suggest that while SHH signaling was found to be essential for *M. bovis* BCG-induced CD4^+^CD25^+^FoxP3^+^ Treg expansion, NOTCH signaling suppress Treg expansion.

### *M. bovis* BCG-responsive SHH signaling is PI3K-mTOR-NF-κB pathway dependent

**Figure 3:**
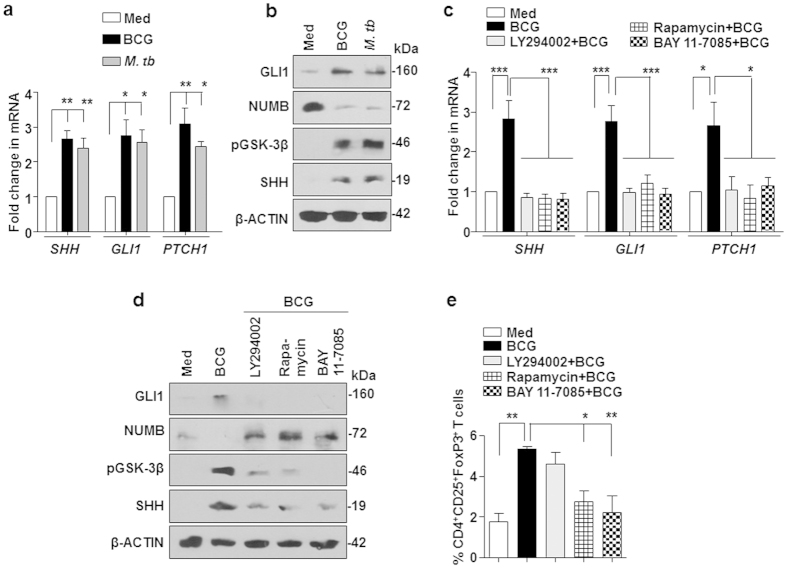
PI3K-mTOR-NF-κB pathway mediates *M. bovis* BCG-induced SHH signaling and Treg expansion. (**a,b**) Five-day-old differentiated immature DCs were infected with 1:10 MOI of *M. bovis* BCG or *M. tuberculosis* H37Ra for 6 h. Transcript (**a**) and protein (**b**) levels of various SHH signaling markers were determined using quantitative real time RT-PCR and immunoblotting respectively. (**c,d**) Expression analysis of SHH signaling markers in immature DCs pretreated with the indicated pharmacological inhibitors for 1 h prior to 6 h infection with *M. bovis* BCG. (**e**) DCs infected with *M. bovis* BCG alone or after pretreatment of LY294002 (PI3K inhibitor), Rapamycin (mTOR inhibitor) or BAY 11-7085 (NF-κB inhibitor) were co-cultured with autologous CD4^+^ T cells. Percentage of CD4^+^CD25^+^FoxP3^+^ T cells (mean ± SEM, n = 4) were analyzed by flow cytometry. All RT-PCR data represents the mean ± SEM from at least 3 independent experiments and all blots are representative of 3 independent experiments. Images have been cropped for presentation; full-size blot is shown in Supplementary Fig. S1. Med, Medium. *P < 0.05; **P < 0.005; ***P < 0.001 (one-way ANOVA followed by Turkey’s multiple-comparisons test).

To further understand the molecular mechanism involved in *M. bovis* BCG-mediated SHH signaling, the activation status of SHH signaling in the DCs was assessed. Stimulation of DCs with *M. bovis* BCG or *M. tuberculosis* induced significant activation of SHH signaling pathway (Fig. 3a,b). Activation of canonical SHH pathways is marked by elevated transcripts of *SHH, GLI1* and *PTCH1* and increased levels of SHH, GLI1, pGSK-3β (Ser9) and decreased NUMB expression. Mycobacteria-stimulated activation of SHH signaling was found to be dependent on PI3K-mTOR-NF-κB pathway as pretreatment of DCs with specific inhibitors of PI3K (LY294002), mTOR (Rapamycin) and NF-κB (BAY 11-7085) led to significant reduction of *M. bovis* BCG-induced SHH signaling (Fig. 3c,d). Corroborating these results, DCs pretreated with mTOR or NF-κB specific inhibitors failed to expand CD4^+^CD25^+^FoxP3^+^ Treg cells (Fig. 3e). However, PI3K-specific inhibitor had no effect on *M. bovis* BCG-induced CD4^+^CD25^+^FoxP3^+^ Treg cells expansion (Fig. 3e).

### SHH signaling-mediated expression of PD-L1 and COX-2-PGE_2_ is required for Treg expansion

**Figure 4:**
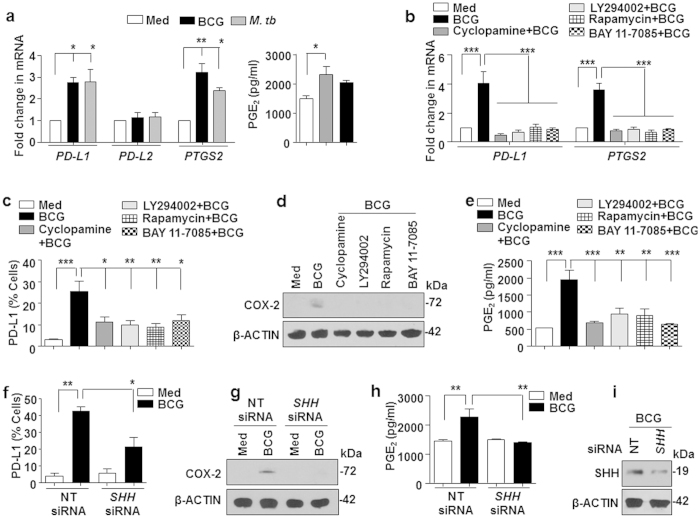
*M. bovis* BCG-induced PD-L1 and COX-2 are SHH signaling dependent. (**a,b**) Quantitative real time RT-PCR for expression analysis of *PD-L1, PD-L2, PTGS2*/COX-2 and ELISA from cell-free supernatants to estimate the secretion of PGE_2_ on (**a**) infection of immature DCs with *M. bovis* BCG or *M. tuberculosis* H37Ra for 12 h or (**b**) infection of indicated pharmacological inhibitor treated immature DCs with *M. bovis* BCG for 12 h. (**c**) DCs were cultured in GM-CSF and IL-4 alone (Med) or with pharmacological inhibitor followed by *M. bovis* BCG as indicated for 24 h. Surface expression of PD-L1 as % positive cells was analyzed by flow cytometry (mean ± SEM, n = 8). (**d,e**) DCs were treated as explained above. Immunoblotting for COX-2 from total cell lysate (**d**) and ELISA for measuring PGE_2_ in the cell-free supernatant (**e**). (**f–i**) Immature human DCs were transiently transfected with NT or *SHH* siRNA. 48 h post transfection, cells were infected with *M. bovis* BCG for 24 h to assess the surface expression of PD-L1 by flow cytometry (mean ± SEM, n = 4) (f) or 12 h to estimate COX-2 protein by immunoblotting (**g**) and PGE_2_ in the cell-free supernatant by ELISA (**h**). Validation of *SHH* siRNA was performed by immunoblotting for SHH in the siRNA-transfected DCs (**i**). All RT-PCR and ELISA data represents the mean ± SEM from at least 3 independent experiments and all blots are representative of 3 independent experiments. Images have been cropped for presentation; full-size blot is shown in Supplementary Fig. S1. Med, Medium; NT, Non-targeting. *P < 0.05; **P < 0.005; ***P < 0.001 (one-way ANOVA followed by Turkey’s multiple-comparisons test).

**Figure 5:**
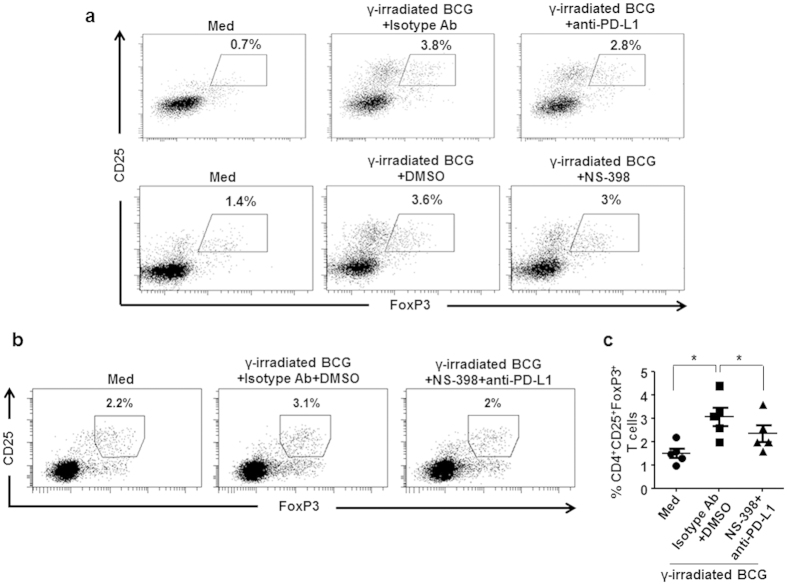
PD-L1 and COX-2 mediate *M. bovis* BCG-induced Treg expansion. (**a**) Inhibition of either PD-L1 or COX-2, partially inhibit the BCG-induced Treg expansion. γ-irradiated BCG-stimulated DCs were incubated with anti-PD-L1 blocking antibody or isotype antibody (upper panel). Alternatively, DCs were pretreated with DMSO or NS-398 (COX-2 inhibitor) before stimulation with BCG (lower panel). After extensive wash, DCs were co-cultured with autologous CD4^+^ T cells for five days. Representative dot-plots showing frequency of CD4^+^CD25^+^FoxP3^+^ Tregs were presented. The Treg response induced by DCs cultured in medium alone is represented by ‘Med’. (**b,c**) Inhibition of both PD-L1 and COX-2 in DCs significantly suppress the ability of BCG to expand Tregs. DCs were pretreated with DMSO or NS-398 before stimulation with BCG. After extensive washing, these DCs were incubated with anti-PD-L1 blocking antibody or isotype antibody and co-cultured with autologous CD4^+^ T cells for five days. CD4^+^CD25^+^FoxP3^+^ Tregs were analyzed by flow cytometry. Representative dot-plots showing frequency of CD4^+^CD25^+^FoxP3^+^ Tregs were presented (**b**). (**c**) Percentage of CD4^+^CD25^+^FoxP3^+^ cells in the DC-CD4^+^ T cell co-cultures (mean ± SEM, n = 5). *P < 0.05 (one-way ANOVA followed by Holm-Sidak’s multiple comparisons test).

Having established that mycobacteria activate the PI3K-mTOR-NF-κB-SHH signaling cascade in human DCs to induce Treg expansion, we attempted to identify the molecular regulators that mediate the process. Utilizing cues from previous investigations^15^,^16^,^17^, we analyzed the role for co-stimulatory molecules PD-L1, PD-L2 and a soluble factor such as COX-2-PGE_2_ in the current study. Stimulation of DCs with *M. bovis* BCG and *M. tuberculosis* resulted in increased expression of *PD-L1* and *PTGS2*/COX-2 (Fig. 4a, left panel). In line with our previous results, PD-L2 was not induced in stimulated DCs. Further, enhanced COX-2 expression was also associated with concomitant increase in PGE_2_ secretion by DCs (Fig. 4a, right panel). Following the previous results, expression of PD-L1 and *PTGS2*/COX-2 transcripts was found to be PI3K-mTOR-NF-κB-SHH dependent (Fig. 4b). Further, surface expression of PD-L1 (Fig. 4c), expression of COX-2 in the whole cell lysate (Fig. 4d) and secretion of PGE_2_ by DCs (Fig. 4e) followed the similar trend. SHH-mediated expression of these mediators was confirmed by reduced expression of PD-L1, COX-2 and PGE_2_ on *M. bovis* BCG infection in the presence of *SHH*-specific siRNA (Fig. 4f–i). Finally, inhibition of either PD-L1 or COX-2 by specific blocking antibody (anti-PD-L1) or pharmacological inhibitor (NS-398, COX-2 inhibitor) could partially inhibit the *M. bovis* BCG-induced Treg expansion (Fig. 5a). Of note, inhibition of both PD-L1 and COX-2 in DCs significantly suppressed the ability of mycobacteria to expand Tregs (Fig. 5b,c).

**Figure 6:**
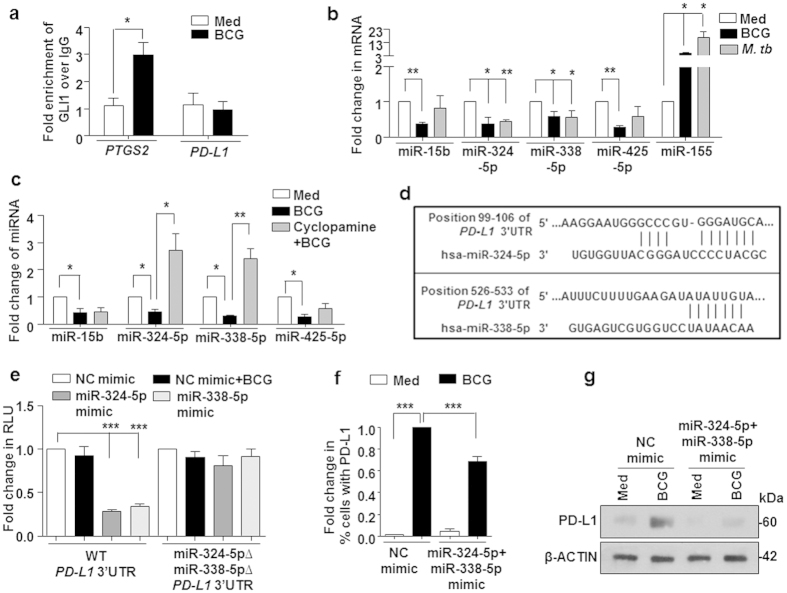
Bi-functional role of SHH signaling to regulate PD-L1 and COX-2 expression. (**a**) The recruitment of GLI1 at human *PTGS2* and *PD-L1* promoter upon infection with *M. bovis* BCG for 12 h in immature DCs was evaluated by ChIP assay. (**b,c**) DCs were infected with *M. bovis* BCG or *M. tuberculosis* H37Ra alone (**b**) or with pharmacological inhibitors of SHH pathway (**c**) and quantitative real time RT-PCR analysis was performed on total RNA isolated using indicated miRNA-specific primers. (**d**) Putative miR-324-5p and miR-338-5p binding sites in the 3′UTR of *PD-L1.* (**e**) THP-1 cells were transfected with WT *PD-L1* 3′UTR or miR-324-5pΔ miR-338-5pΔ *PD-L1* 3′UTR constructs with miR-324-5p or miR-338-5p mimics as indicated. Transfected THP-1 cells were further stimulated with *M. bovis* BCG as indicated and luciferase assay was performed. (**f,g**) DCs transfected with control or miR-324-5p and miR-338-5p mimics were infected with *M. bovis* BCG for 24 h (**f**) or 18 h (**g**) as indicated. While surface expression of PD-L1 was evaluated by flow cytometry (mean ± SEM, n = 3) (**f**), protein levels of PD-L1 in the total cell lysate was assessed by immunoblotting (**g**). All RT-PCR and luciferase data represents the mean ± SEM from 3 independent experiments. Images have been cropped for presentation; full-size blot is shown in Supplementary Fig. S1. Med, Medium; NC, Negative control. *P < 0.05; **P < 0.005; ***P < 0.001 (Student’s *t*-test for Panel a, one-way ANOVA followed by Turkey’s multiple-comparisons test for others).

GLI1, a zinc-finger protein, is a dedicated transcription factor for SHH signaling-mediated gene expression^31^. ChIP analysis revealed *M. bovis* BCG-induced recruitment of GLI1 to the promoter of *PTGS2*/COX-2 in DCs suggesting a SHH-dependent transcriptional regulation of COX-2 expression (Fig. 6a). However, no apparent change was observed on *PD-L1* promoter (Fig. 6a). In this context, we speculated the role for modulation of negative regulators of PD-L1 by infection-responsive SHH signaling. MiRNAs belong to one such class of post-transcriptional regulators. Extensive bioinformatic analysis (TargetScan, miRanda, miRWalk and RNAhybrid) together with available cues on downregulated miRNAs in tuberculosis patient^20^,^32^ or *M. tuberculosis* or *M. bovis* BCG samples^33^ identified miR-15b, miR-324-5p, miR-338-5p and miR-425-5p as candidate miRNAs that could target PD-L1. In line with this, DCs infected with *M. tuberculosis* or *M. bovis* BCG exhibited reduced levels of the identified miRNAs. MiR-155 was utilized as a known positive control (Fig. 6b). However, among the *M. bovis* BCG-downregulated miRNAs, expression of miR-324-5p and miR-338-5p was found to be SHH-regulated as the downregulation of miR-324-5p and miR-338-5p was significantly rescued in presence of a SHH inhibitor, cyclopamine (Fig. 6c). The target sites located at the residues spanning from 99–106 (for miR-324-5p) and 526–533 (for miR-338-5p) of the 3′UTR of *PD-L1* were identified as critical for miRNA-3′UTR interactions (Fig. 6d). To establish that *PD-L1* is the bonafide targets of miR-324-5p and miR-338-5p, we utilized the classical 3′UTR luciferase assays. Transfection of a monocytic cell line THP-1 with miR-324-5p or miR-338-5p mimics markedly reduced WT *PD-L1* 3′UTR luciferase activity. However, no significant reduction was observed when mutant construct for miR-324-5p and miR-338-5p binding on *PD-L1* 3′UTR was utilized (Fig. 6e). These results thus validate that *PD-L1* is a direct target of miR-324-5p and miR-338-5p. In accordance with this observation, we found that DCs expressing miR-324-5p or miR-338-5p miRNAs displayed reduced ability to induce the surface expression as well as total protein levels of PD-L1 on *M. bovis* BCG infection (Fig. 6f,g). Together, these results highlight a dichotomous role for *M. bovis* BCG-induced SHH signaling in DCs during expression of COX-2 and PD-L1.

### NOTCH1 signaling-dependent PI3K-mTOR-NF-κB axis regulates the Th1 responses

**Figure 7:**
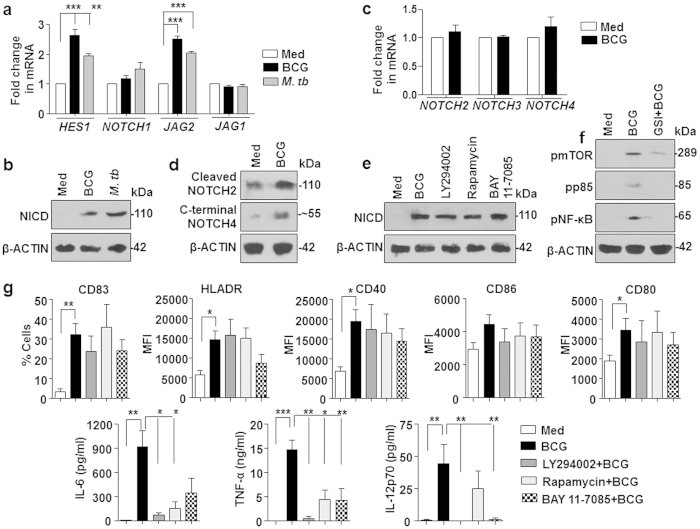
*M. bovis* BCG-induced NOTCH1 signaling in DCs regulates PI3K-mTOR-NF-κB pathway. (**a**) Quantitative real time RT-PCR for assessing NOTCH1 signaling markers, *HES1, NOTCH1, JAG1* and *JAG2* on infection of immature DCs with *M. bovis* BCG or *M. tuberculosis* H37Ra for 6 h. (**b**) Activation of NOTCH1 signaling was determined by immunoblotting for NICD in DCs infected with *M. bovis* BCG or *M. tuberculosis* H37Ra. (**c,d**) Transcript (**c**) and intracellular domain (**d**) of NOTCH2-4 on *M. bovis* BCG stimulation was analyzed by quantitative real time RT-PCR (**c**) or immunoblotting (**d**). (**e**) NOTCH1 signaling activation was assessed by immunoblotting for NICD in DCs pretreated with the indicated pharmacological inhibitors and infected with *M. bovis* BCG. (**f**) Immunoblotting for evaluating PI3K-mTOR-NF-κB pathway activation using DCs infected with *M. bovis* BCG with or without GSI (NOTCH signaling inhibitor). (**g**) DCs were cultured with GM-CSF and IL-4 and left untreated (Med) or infected with *M. bovis* BCG alone or after 1 h pretreatment with the indicated inhibitors for 24 h. Surface expression of maturation markers CD83, HLA-DR, CD40, CD86 and CD80 were examined by flow cytometry (mean ± SEM, n = 7–10). Data is represented as % positive cells or MFI ((**g**), top panels). Cell-free supernatants from the above-said experiment were assessed for secretion of IL-6, TNF-α, IL-12p70 by cytokine bead array ((**g**), lower panels) (mean ± SEM, n = 5–7). All RT-PCR data represents the mean ± SEM from at least 3 independent experiments and all blots are representative of 3 independent experiments. Images have been cropped for presentation; full-size blot is shown in Supplementary Fig. S2. Med, Medium. *P < 0.05; **P < 0.005; ***P < 0.001 (one-way ANOVA followed by Turkey’s multiple-comparisons test).

After establishing the role for SHH signaling, the other identified signaling pathway (Fig. 1a,b) in DCs that affected the Treg expansion, NOTCH signaling, was analyzed. NOTCH1 signaling activation in DCs-infected with mycobacteria was assessed using transcript analysis of *HES1, NOTCH1, JAG1* and *JAG2* and generation of NICD. Elevated levels of *HES1* and *JAG2* transcripts and NICD marked the activation of NOTCH1 signaling on *M. tuberculosis* or *M. bovis* BCG infection (Fig. 7a,b). Exploring the role for other NOTCH receptor, it was found that while no increase in the transcripts of *NOTCH2-4* was observed on *M. bovis* BCG stimulation, intracellular domain of NOTCH2 and NOTCH4 were induced on mycobacterial infection (Fig. 7c,d). However, we chose to analyze the function of NOTCH1 signaling in the current study as its activation was found comparatively more robust. While inhibition of PI3K-mTOR-NF-κB axis did not alter the *M. bovis* BCG-induced NICD generation (Fig. 7e), pharmacological intervention of NOTCH1 signaling using GSI significantly abrogated the *M. bovis* BCG-mediated activation of PI3K-mTOR-NF-κB pathway suggesting that NOTCH1 pathway regulates the PI3K-mTOR-NF-κB signaling cascade (Fig. 7f). In line with this observation, NOTCH1-responsive PI3K-mTOR-NF-κB axis was found essential for generation of *M. bovis* BCG-induced inflammatory cytokines like IL-6, TNF-α and IL-12 despite for the fact that no change in the infection-induced DC maturation was observed when PI3K-mTOR-NF-κB pathway was inhibited (Fig. 7g).

### Counter-regulation of SHH and NOTCH1 signaling during *M. bovis* BCG infection

**Figure 8:**
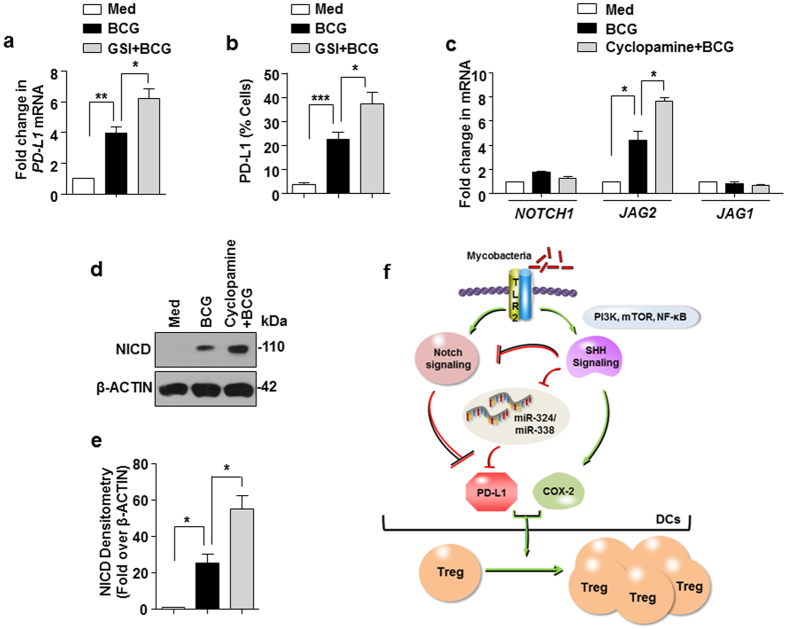
Counter-regulation of NOTCH1 and SHH pathway functions during *M. bovis* BCG infection of DCs. (**a,b**) Transcript (**a**) and surface (**b**) expression of PD-L1 in DCs infected with *M. bovis* BCG alone or after 1 h pretreatment of GSI by quantitative real time RT-PCR and flow cytometry (mean ± SEM, n = 7) respectively. (**c–e**) Immature DCs were pretreated with SHH signaling inhibitors and infected with *M. bovis* BCG for 6 h. NOTCH1 signaling markers, *NOTCH1, JAG1* and *JAG2* transcripts were analyzed using quantitative real time RT-PCR (**c**), NICD by immunoblotting (**d**) and densitometric analysis of panel D (**e**). (**f**) Model: schematic representation of the obtained results. All RT-PCR and densitometry data represents the mean ± SEM from at least 3 independent experiments and all blots are representative of 3 independent experiments. Images have been cropped for presentation; full-size blot is shown in Supplementary Fig. S2. Med, Medium. *P < 0.05; **P < 0.005; ***P < 0.001 (one-way ANOVA followed by Turkey’s multiple-comparisons test).

Previously, we found that NOTCH1 signaling negatively regulated the *M. bovis* BCG-mediated CD4^+^CD25^+^FoxP3^+^ Treg expansion (Fig. 1a,b). In accordance with this observation, DCs treated with GSI exhibited increased expression of PD-L1 on *M. bovis* BCG infection (Fig. 8a,b). This suggests that *M. bovis* BCG-induced NOTCH1 signaling negatively regulates PD-L1 expression to regulate Treg expansion. On the other hand, DCs treated with cyclopamine displayed enhanced expression of *JAG2* transcript (Fig. 8c) and increased NICD formation (Fig. 8d,e). Together, these results indicate a counter-balance between the effects of infection-induced NOTCH1 and SHH signaling cascades to regulate CD4^+^CD25^+^FoxP3^+^ Treg expansion.

## Discussion

Induction and expansion of an inhibitory CD25^+^FoxP3^+^ Treg population reckons as one of the immune evasion strategies that mycobacteria employ to combat the protective immune responses^5^,^9^,^15^,^16^. In the current investigation, we found novels roles for SHH and NOTCH1 pathways in modulating *M. bovis* BCG-induced Treg expansion. We attribute the current observation to Treg expansion and not Treg induction as co-culture experiments with naïve CD4^+^ T did not show significant change in Treg population on mycobacterial infection (data not shown).While no previous reports were available implicating SHH signaling regulating Treg functions, multiple studies in mice and humans have identified NOTCH signaling to modulate Treg population. Overexpression of JAG1 signaling in DCs induced a regulatory phenotype in CD4^+^ T cells in both humans and mice^34^,^35^. However, direct role of JAG1 assisting the differentiation of naive CD4^+^ T to Tregs is not established, underscoring the differential functions of JAG1 in regulating Treg generation in humans and mice^36^. In another investigation, NOTCH1 signaling was found to promote TGF-β-mediated Treg functions in both humans and mice^26^. On the contrary, DLL4-mediated NOTCH signaling inhibits TGF-β-mediated Treg development and JNK-responsive STAT5 activation, a requisite for FoxP3 expression and maintenance^25^,^37^. In line with latter observation, mycobacteria-responsive activation of NOTCH1-JAG2 axis in DCs was found to suppress CD25^+^FoxP3^+^ Treg population. Supporting our observation, while JAG2 was found to expand Tregs during graft rejection in mice^38^, elevated JAG2 levels in hematopoietic progenitor cells was required for Treg expansion in mice to suppress T cell-mediated diseases^39^. Significant contribution of the WNT pathway in Treg expansion was not observed. However, previous reports suggest both positive and negative regulation of Treg functions by the canonical WNT signaling pathway. While inhibition of β-CATENIN in DCs comprised their ability to induce Tregs^27^, WNT signaling activation inhibited the transcriptional activity of FoxP3 in T cells^28^.

*M. bovis* BCG-induced NOTCH1-PI3K-mTOR-NF-κB signaling in DCs was also found to promote inflammatory cytokines like IL-1β, IL-6, TNF-α and IL-12. Multiple investigations in both humans and mice have subscribed a NOTCH signaling ligand-specific T cell differentiation^40^: Th1^41^,^42^,^43^, Th2^44^,^45^,^46^ and Th17^47^,^48^. However, in the current investigation, mycobacterial infection of human DCs induced NOTCH1 signaling to program the cells towards pro-inflammatory in nature. This observation is in accordance with our previous report that suggested a function of a mycobacterial immunodominant protein, Rv0754, in regulating a NOTCH1-PI3K pathway-dependent pro-inflammatory environment to subvert CTLA-4- and TGF-β-induced suppression of DC maturation^22^. Interestingly, though infection-induced signaling pathways regulated the DC functions in terms of modulating the specific cytokines and T cell phenotype, no apparent role for these identified pathways was found for DC maturation.

To identify the molecular mechanism that mediates SHH and NOTCH signaling-responsive T cell phenotype during mycobacterial infection, we chose to analyze the contributions of PD-L1, PD-L2 and PGE_2_. The co-stimulatory molecules on the APCs, PD-L1 and PD-L2 signal to the PD-1 on the T cells to orchestrate a Treg differentiation and expansion^49^. Formation of the PD-L1 and PD-1 ligand-receptor complex triggers the SHP1/2 activation that suppresses the STAT1 activity, thereby abrogating IFN-γ-mediated responses. STAT1 is otherwise known to inhibit FoxP3 expression^50^. Importantly, reports suggest that induced expression of PD-L1 in human DCs is necessary for mycobacteria-induced Treg expansion^15^,^16^. However, PD-L1 KO and PD-1 KO mice strangely displayed exacerbated tuberculosis disease with excessive inflammatory responses and increased susceptibility to infection underscoring the crucial role for PD-L1 signaling during mycobacterial infection^14^,^51^. Interestingly, inhibition of PD-L1 in DCs also promoted mycobacteria-induced IFN-γ production in T cells^52^. Of note, COX-2 catalyzed PGE_2_ serves as a cue for Treg expansion and functions^53^,^54^. PGE_2_-EP signaling is known to aid in FoxP3 expression^55^. Importantly, in humans, mycobacterial infection triggers PGE_2_-dependent expansion of CD25^+^FoxP3^+^ Tregs^17^. In agreement with all these observations, our results revealed that SHH-dependent expression of PD-L1 and COX-2-PGE_2_ during mycobacterial infection induces the Treg expansion. It was also noted that there was synergistic effect of PD-L1 and COX-2 in mediating mycobacteria-induced Treg expansion. In fact, upon inhibition of both PD-L1 and COX-2, the Treg frequency reached close to medium control conditions. Interestingly, a recent report showed similar synergistic effects of COX and PD-1 for eradication of tumors and its usefulness as adjuvants for immune-based therapies^56^. Further, while expression of COX-2 was a transcriptional regulation by SHH signaling, post-transcriptional regulation of PD-L1 by SHH signaling-regulated miRNAs was observed.

Based on available information on genome-wide miRNA profiling in tuberculosis patients vs healthy individuals^20^,^32^,^57^,^58^ and *ex-vivo* infection studies^33^, a panel of miRNAs that were downregulated on mycobacterial infection and served as putative miRNAs that target PD-L1 were chosen for the study. MiR-155 was utilized as a positive control as it is not only known to be induced to several manifolds during mycobacterial infection, but also associated with maturation of DCs^59^,^60^. Among the tested miRNAs, miR-324-5p and miR-338-5p were identified as the bonafide miRNAs that target PD-L1 in a SHH-dependent manner and hence, downregulated during mycobacterial infection. However, the mechanism of SHH-mediated transcriptional regulation of miR-324-5p and miR-338-5p needs to be studied further.

Mycobacteria harbors numerous antigens that are recognized by several pattern recognition receptors; TLR2 being the dominant one. Various studies, including ours, have suggested that mycobacteria and its antigens induce TLR2 signaling in both macrophages and DCs^16^,^18^,^19^,^21^,^22^,^23^,^57^,^61^. Importantly, many of these previous studies have implicated that mycobacteria-induced activation of NOTCH in both macrophages^18^,^62^ and DCs^22^ and mycobacteria-induced activation of SHH pathways in macrophages^20^,^21^ were TLR2-dependent. Further, while current and previous results^18^,^22^ suggest that mycobacteria-induced NOTCH1 signals the activation of PI3K-mTOR-NF-κB axis, current and a previous study^20^ also suggests that mycobacteria-induced SHH signaling is PI3K-mTOR-NF-κB dependent. Keeping these results in mind, TLR2-dependent activation of NOTCH1 signaling during mycobacterial infection could play a dominant role in DCs. However, we have not assessed the role of TLR2 in the current study.

In summary, establishing novel roles for SHH and NOTCH1 signaling pathways, we found mycobacteria-activated SHH signaling induces PD-L1 and COX-2-PGE_2_ to mediate the expansion of CD4^+^CD25^+^FoxP3^+^ Tregs whereas infection-induced NOTCH1 signaling was found to suppress the Treg expansion (Fig. 8f). Thus, mycobacteria modulate the host signaling pathways and molecular regulators in DCs to determine the functional outcome of the immune responses including Tregs expansion.

## Methods

### Antibodies

Fluorescein isothiocyanate (FITC)-conjugated monoclonal antibodies (mAbs) to CD86, CD1a, PD-L1 (CD274), IFN-γ (all anti-human), phycoerythrin (PE)-conjugated mAbs to CD80, CD83 and CD25, APC-conjugated HLA-DR (all anti-human), anti-human Alexa Fluor 700-CD4 were from BD Biosciences, and PE-conjugated mAb to anti-human CD40 was from Becton Dickinson. Human Foxp3-APC, anti-PD-L1 and isotype control were from eBioscience. CD14 magnetic beads, T cell isolation kit II, GM-CSF and IL-4 were from Miltenyi Biotec. Anti-β-ACTIN and anti-PGE_2_ antibodies were purchased from Sigma-Aldrich. Anti-SHH, anti-GLI1, anti-NUMB, anti-Ser9 phospho-GSK-3β, anti-NICD (Cleaved Notch1), anti-Ser2448 phospho mTOR, anti-Tyr458 phospho p85 and anti-Ser536 phospho NF-κB p65 were purchased from Cell Signaling Technology. Anti-COX-2 was from Calbiochem. Anti-Notch 2 intracellular domain and anti-Notch 4 -C-terminal antibodies were from Abcam. HRP conjugated anti-rabbit IgG and anti-mouse IgG was obtained from Jackson ImmunoResearch.

### Generation of Human DCs

Human Peripheral blood mononuclear cells (PBMC) were isolated from buffy coats of the healthy blood donors purchased from Centre Necker-Cabanel, Etablissement Français du Sang, Paris, France. Ethical committee permission was obtained for the use of buffy bags of healthy donors (N°12/EFS/079). All samples were analyzed anonymously. Circulating monocytes were isolated from these PBMCs using CD14 magnetic beads. The purity was more than 98%. Monocytes were cultured in RMPI-1640 medium containing 10% FCS in the presence of GM-CSF (1000 IU/10^6^ cells) and IL-4 (500 IU/10^6^ cells) for 5 days to obtain immature DCs and used for subsequent experiments.

### Stimulation of DCs with mycobacteria

0.5 × 10^6^ immature DCs (FACS, Co-culture experiments) or 10^6^ immature DCs (Immunoblotting, RNA isolation, transfections experiments) or 2 × 10^6^ immature DCs (ChIP experiments) were washed and infected with 1:10 multiplicity of infection (MOI) of *M. bovis* BCG or *M. tuberculosis* H37Ra for indicated time in the presence of GM-CSF and IL-4. Supernatants were collected for analyzing cytokines and cells were utilized for further indicated assays. *M. bovis* BCG Pasteur 1173P2 was obtained from Pasteur Institute, Paris, France and *M. tuberculosis* H37Ra was kind research gift from Dr. P. Ajitkumar, IISc, India. Mycobacteria were grown in Middlebrook 7H9 broth to mid-log phase and then aliquoted, following which it was stored at −70 °C. Representative vials were thawed and the mycobacterial cells’ viability was then assessed by plating on Middlebrook 7H10 agar plates.

### Treatment with pharmacological reagents

Cells were treated with the given inhibitor (from Calbiochem) for 1 h before experimental treatments at following concentrations: Cyclopamine (15 μM); IWP-2 (5 μM); GSI (750 nM); LY294002 (10 μM); Rapamycin (200 nM); BAY 11-7085 (5 μM); NS-398 (25 μM). DMSO at 0.1% concentration was used as the vehicle control. In all experiments involving pharmacological reagents, a tested concentration was used after careful titration experiments assessing the viability of the DCs using the MTT (3-(4,5-Dimethylthiazol-2-yl)-2,5-diphenyltetrazolium bromide) assay.

### Transient transfection studies

Transient transfection of immature DCs with 100 nM siRNA or 150 nM miRNA mimics were carried out utilizing Oligofectamine reagent (Life Technologies). *SHH*, non-targeting siRNA and siGLO Lamin A/C were obtained from Dharmacon as siGENOME™ SMARTpool reagents, which contain a pool of four different double-stranded RNA oligonucleotides. MiR-324-5p, miR-338-5p mimics and negative control mimics were purchased from Ambion (Life Technologies). Transfection efficiency was found to be more than 50% in all the experiments as determined by counting the number of siGLO Lamin A/C positive cells in a microscopic field using fluorescent microscope. 48 h post siRNAs transfection or 24 h post miRNAs transfection, the cells were treated or infected as indicated and processed for analysis.

### RNA isolation and quantitative real time RT-PCR

DCs were treated or infected as indicated and total RNA from the cells was isolated by TRI reagent (Sigma-Aldrich). 2 μg of total RNA was converted into cDNA using First strand cDNA synthesis kit (Invitrogen). Quantitative real time RT-PCR was performed using SYBR Green PCR mixture (KAPA Biosystems) for quantification of the target gene expression. All the experiments were repeated at least three times independently to ensure the reproducibility of the results. *GAPDH* was used as internal control. The primers used for PCR amplification are: *GAPDH* forward 5′-ggagcgagatccctccaaaat-3′, *GAPDH* reverse 5′-ggctgttgtcatacttctcatgg-3′; *PD-L1* forward 5′-ggacaagcagtgaccatcaag-3′, *PD-L1* reverse 5′-cccagaattaccaagtgagtcct-3′; *PD-L2* forward 5′-accgtgaaagagccactttg-3′, *PD-L2* reverse 5′-gcgaccccatagatgattatgc-3′; *PTGS2* forward 5′-ggtggagaagtgggttttca-3′, *PTGS2* reverse 5′-gactcctttctccgcaacag-3′; *SHH* forward 5′-ctcgctgctggtatgctcg-3′, *SHH* reverse 5′-atcgctcggagtttctggaga-3′; *GLI1* forward 5′-agcgtgagcctgaatctgtg-3′, *GLI1* reverse 5′-cagcatgtactgggctttgaa-3′; *PTCH1* forward 5′-ccagaaagtatatgcactggca-3′; *PTCH1* reverse 5′-gtgctcgtacatttgcttggg-3′; *HES1* forward 5′-acacgacaccggataaaccaa-3′, *HES1* reverse 5′-gccgccagctatctttcttca-3′; *NOTCH1* forward 5′-gaggcgtggcagactatgc-3′, *NOTCH1* reverse 5′-cttgtactccgtcagcgtga-3′; *JAG1* forward 5′-tcgggtcagttcgagttgga-3′, *JAG1* reverse 5′-aggcacactttgaagtatgtgtc-3′; *JAG2* forward 5′-tgggactgggacaacgatac-3′, *JAG2* reverse 5′-agtggcgctgtagtagttctc-3′; *NOTCH2* forward 5′-tattgatgactgccctaaccaca-3′, *NOTCH2* reverse 5′-atagcctccattgcggttgg-3′; *NOTCH3* forward 5′-aagtcggggcacagtttctc-3′, *NOTCH3* reverse 5′-ctcccactcaccgatctgg-3′; *NOTCH4* forward 5′-gatgggctggacacctacac-3′, *NOTCH4* reverse 5′-cacacgcagtgaaagctacca-3′.

### Quantification of miRNA expression

For detection of miRNAs, total RNA was isolated from infected or treated DCs using the TRI reagent. Quantitative real time RT-PCR for miR-155, miR-15b, miR-324-5p, miR-338-5p and miR-425-5p was performed using TaqMan miRNA assays (Ambion) as per manufacturer’s instructions. U6 snRNA was used for normalization.

### Immunoblotting

Infected or treated DCs were lysed in RIPA buffer constituting 50 mM Tris-HCl (pH 7.4), 1% NP-40, 0.25% Sodium deoxycholate, 150 mM NaCl, 1 mM EDTA, 1 mM PMSF, 1 μg/ml of each aprotinin, leupeptin, pepstatin, 1 mM Na_3_VO_4_ and 1 mM NaF. Equal amount of protein from each cell lysate was resolved on a 12% SDS-polyacrylamide gel and transferred to polyvinylidene difluoride membranes (PVDF) (Millipore) by the semi-dry transfer (Bio-Rad) method. Nonspecific binding was blocked with 5% nonfat dry milk powder in TBST [20 mM Tris-HCl (pH 7.4), 137 mM NaCl, and 0.1% Tween 20] for 60 min. The blots were incubated overnight at 4 °C with primary antibody followed by incubation with anti-rabbit-HRP or anti-mouse-HRP secondary antibody in 5% BSA for 2 h. After washing in TBST, the immunoblots were developed with enhanced chemiluminescence detection system (Perkin Elmer) as per manufacturer’s instructions. β-ACTIN was used as loading control. For probing another protein in the same region of the PVDF membrane, the blots were stripped in the stripping buffer [62.5 mM Tris-HCl (pH 6.8), 2% SDS and 0.7% β-mercaptoethanol] at 60 °C on a shaker, blocked with 5% nonfat dry milk powder and probed with antibodies as mentioned above.

### DC:CD4^+^ T cell co-cultures

Autologous CD4^+^ T cells were obtained using T cell isolation kit II. The treated DCs were co-cultured with CD4^+^ T cells at 1:10 for 5 days. Cell-free culture supernatants were collected for the analysis of T cell cytokines. Cells were treated with PMA-Ionomycin and Golgistop for 5 h. Following this, the T cells were processed for staining with fluorchrome-conjugated mAbs for flow cytometry.

### γ-irradiated BCG-stimulated DC:CD4^+^ T cell co-culture

Immature DCs treated with DMSO or 25 μM NS-398 for 1 h were stimulated with 20 μg/ml γ-irradiated BCG (Strain AF 2122/97 (ATCC^®^ BAA-935™) obtained through BEI Resources, NIAID, NIH) for 24 h. Post stimulation, DCs were thoroughly washed with RPMI, counted and blocked with isotype control or anti-PD-L1 (10 μg/ml) for 1 h. DCs were then co-cultured with autologous CD4^**+**^ T cells at 1:10 ratio for 5 days. On day 0 and day 3 of the co-culture, 25 μM NS-398 was added for efficient COX-2 inhibition. T cells were processed for staining with fluorchrome-conjugated mAbs for flow cytometry.

### Flow cytometry

Surface staining of CD1a, HLA-DR, CD40, CD83, CD80 and CD86 on DCs was performed using fluorochrome-conjugated mAbs. T cells were surface-labeled with CD25- and CD4-specific fluorochrome-conjugated mAbs and then washed, fixed, permeabilized for intracellular staining of FoxP3. Cells were further processed for flow cytometry wherein 5000 gated events were recorded for each sample acquired in BD LSR II or BD FACSCanto II and the data was analyzed using FACSDiva™ software (BD Biosciences).

### Cytokine analysis

Cytokines were quantified in the cell-free culture supernatants using BD™ Cytometric Bead Array (CBA) human inflammatory cytokine kits (IL-1β, CXCL8/IL-8, TNF-α, IL-6, IL-10 and IL-12p70) and human Th1/Th2 cytokine kits (IL-2, IL-4, IL-5, IL-10, TNF-α and IFN-γ) (BD Biosciences). The data were analyzed using FCAP Array™ software (BD Biosciences).

### Enzyme immunoassay for PGE_2_

Enzyme immunoassays for quantitation of PGE_2_ were carried out in 96-well microtiter plates (Nunc) using culture supernatant. ELISA plates were incubated with culture supernatant overnight at 4 °C followed by three washes with 1X PBST. After blocking with 1% BSA for 1 h at 37 °C, wells were incubated with anti-PGE_2_ antibod y for 6 h at 37 °C followed by washing with 1X PBST. The plates were further incubated with HRP conjugated anti-rabbit secondary antibody for 2 h at 37 °C. The assay was developed with 3,3′,5,5′-tetramethylbenzidine (Sigma-Aldrich). The absorbance values were measured at 450 nm by using ELISA reader (Molecular Devices).

### Chromatin Immunoprecipitation (ChIP) Assay

ChIP assays were carried out using a protocol provided by Upstate Biotechnology and Sigma-Adrich with certain modifications. Briefly, DCs were fixed with 1.42% formaldehyde for 15 min at room temperature followed by inactivation of formaldehyde with addition of 125 mM glycine. Nuclei were lysed in 0.1% SDS lysis buffer [50 mM Tris-HCl (pH 8.0), 200 mM NaCl, 10 mM HEPES (pH 6.5), 0.1% SDS, 10 mM EDTA, 0.5 mM EGTA, 1 mM PMSF, 1 μg/ml of each aprotinin, leupeptin, pepstatin, 1 mM Na_3_VO_4_ and 1 mM NaF]. Chromatin was sheared using Bioruptor Plus (Diagenode) at high power for 40 rounds of 30 sec pulse ON/45 sec OFF. Chromatin extracts containing DNA fragments with an average size of 500 bp were immunoprecipitated using specific antibodies or rabbit preimmune sera according to the experiment complexed with Protein A agarose beads (Bangalore Genei). Immunoprecipitated complexes were sequentially washed [Wash Buffer A: 50 mM Tris-HCl (pH 8.0), 500 mM NaCl, 1 mM EDTA, 1% Triton X-100, 0.1% Sodium deoxycholate, 0.1% SDS and protease/phosphatase inhibitors; Wash Buffer B: 50 mM Tris-HCl (pH 8.0), 1 mM EDTA, 250 mM LiCl, 0.5% NP-40, 0.5% Sodium deoxycholate and protease/phosphatase inhibitors; TE: 10 mM Tris-HCl (pH 8.0), 1 mM EDTA] and eluted in elution buffer [1% SDS, 0.1 M NaHCO_3_]. After treating the eluted samples with RNase A and Proteinase K, DNA was precipitated using phenol-chloroform-ethanol method. Purified DNA was analyzed by quantitative real time RT-PCR. All values in the test samples were normalized to amplification of the specific gene in Input and IgG pull down and represented as fold enrichment. All ChIP experiments were repeated at least three times and the primers utilized for GLI1 binding at *PTGS2* promoter are forward 5′-aaattgcgtaagcccggtggg-3′, reverse 5′-gacatctggcggaaacctgtgc-3′ and at *PD-L1* promoter are forward 5′-caggcacggtggctcaagcct-3′, reverse 5′-tctgccaccctaaggattaaggctgcgg-3′.

### *PD-L1* 3′UTR WT/mutant generation and luciferase assay

The 3′UTR of *PD-L1* was PCR amplified and cloned into pmirGLO vector using the restriction enzymes SacI and XbaI. Primer pairs used: WT *PD-L1* 3′UTR forward 5′-cgagctcgcattggaacttctgatcttc-3′, reverse 5′-gctctagagttatagaggagaccaagcac-3′. The miR-324-5p and miR-338-5p binding sites were mutated in *PD-L1* 3′UTR by nucleotide replacements through site-directed mutagenesis using the megaprimer inverse PCR method. The forward primer comprised the desired mutation and respective reverse primer was used to generate megaprimers. Primer pairs used: miR-324-5p megaprimer forward 5′-gcccgtaaagcataggcaat-3′, reverse 5′-cgtcgatgagcccctcagg-3′; miR-338-5p megaprimer forward 5′-tgaagatgcgccacagtagatgttac-3′, reverse 5′-gttatagaggagaccaagcac-3′. The miR-324-5p megaprimer was used to amplify the WT *PD-L1* 3′UTR plasmid to generate miR-324-5pΔ *PD-L1* 3′UTR plasmid. The double mutant plasmid was generated utilizing a megaprimer mutant for miR-338-5p-binding sites on the miR-324-5pΔ mutant *PD-L1* plasmid background. THP-1, human monocytic cell line obtained from the National Center for Cell Sciences, Pune, India, was used for the transfection studies. Cells were cultured in DMEM (Gibco-Life Technologies) containing 10% FBS (Gibco-Life Technologies). Transient transfection of THP-1 cells with 3′UTR constructs, β-Galactosidase construct and the miRNA mimics were performed as indicated using low m.w. polyethylenimine (Sigma-Aldrich). After 48 h of transfection and desired stimulation, cells were lysed in Reporter lysis buffer (Promega) and assayed for luciferase activity using Luciferase Assay Reagent (Promega) as per the manufacturer’s instructions. The results were normalized for transfection efficiencies measured by β-galactosidase activity. O-nitrophenol β-D-galactopyranoside (HiMedia) was utilized for the β-galactosidase assay.

### Statistical analysis

Levels of significance for comparison between samples were determined by the Student’s *t* test distribution and one-way ANOVA. The data in the graphs are expressed as the mean ± S.E for values from 3 independent experiments and *P* values < 0.05 were defined as significant. Graphpad Prism 5.0 software (Graphpad software) was used for all the statistical analysis.

## Additional Information

**How to cite this article**: Holla, S. *et al*. Mycobacteria-responsive sonic hedgehog signaling mediates programmed death-ligand 1- and prostaglandin E_2_-induced regulatory T cell expansion. *Sci. Rep.* **6**, 24193; doi: 10.1038/srep24193 (2016).

## Supplementary Material

Supplementary Information
