# Supplementary material for: Mycobacteria-responsive sonic hedgehog signaling mediates programmed death-ligand 1- and prostaglandin E2-induced regulatory T cell expansion
Source: Sci Rep. 2016 Apr 15;6:24193. doi: 10.1038/srep24193 (PMC4832185; doi:10.1038/srep24193)
Supplement: Supplementary Information [file srep24193-s1.pdf]

Supplementary Information for

Mycobacteria-responsive sonic hedgehog signaling mediates programmed death-ligand 1- and prostaglandin E_2-induced regulatory T cell expansion

Sahana Holla[1], Emmanuel Stephen-Victor[2,3,4], Praveen Prakhar[1], Meenu Sharma[2,3], Chaitrali Saha[2,3,5], Vibha Udupa[1], Srinivas V Kaveri[2,3,4,6], Jagadeesh Bayry[2,3,4,6,*] & Kithiganahalli Narayanaswamy Balaji[1,*]

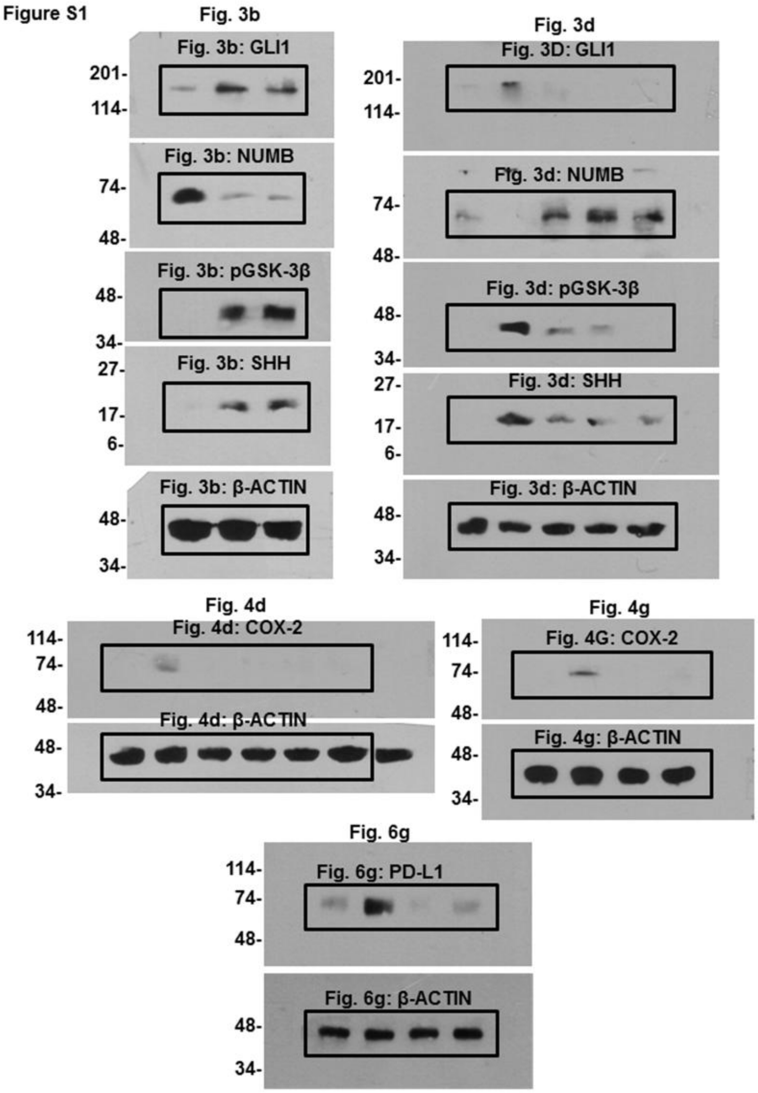

Supplementary Figure S1. Original western blots for images used in Figure 3, 4 and 6. Black boxes indicate the specific bands used in the main figure.

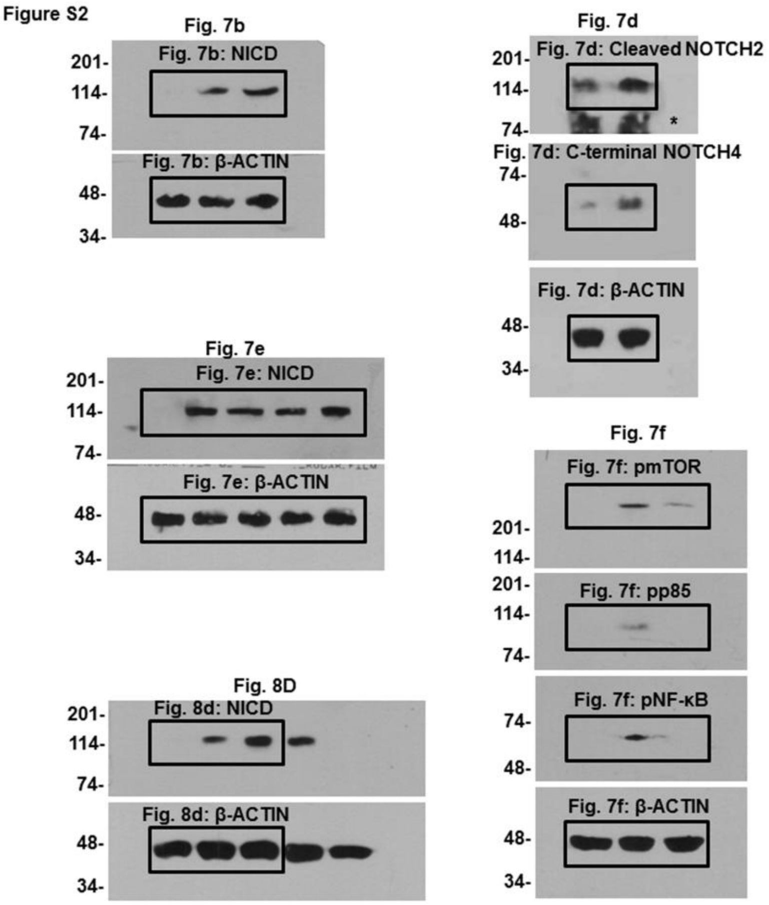

Supplementary Figure S2. Original western blots for images used in Figures 7 and 8. Black boxes indicate the specific bands used in the main figure. * indicates the non-specific bands.
